# Candidemia Among ICU Patients: Species Characterisation, Resistance Pattern and Association With Candida Score: A Prospective Study

**DOI:** 10.7759/cureus.24612

**Published:** 2022-04-29

**Authors:** Shabbir Ahmad, Shailesh Kumar, Kamlesh Rajpal, Richa Sinha, Rakesh Kumar, Sweta Muni, Namrata Kumari

**Affiliations:** 1 Microbiology, Indira Gandhi Institute of Medical Sciences, Patna, IND; 2 Microbiology, Sanjay Gandhi Postgraduate Institute of Medical Sciences, Lucknow, IND

**Keywords:** anti-fungal treatment, anti-fungal susceptibility, candida score, intensive care unit, candida species

## Abstract

Introduction

Candidiasis is a significant cause of morbidity and mortality in immunocompromised patients admitted in intensive care units. Identification of Candida species is essential for effective treatment. However, in absence of proven fungemia, guidelines to initiate therapy are yet to be defined.

Materials and methods

During the study (16 months: September 2018 to December 2019), samples (urine, sputum, blood, tracheal aspirate, urinary catheter) were collected from ICU patients and prospectively evaluated. Microscopy, culture, and antifungal susceptibility testing were performed as per standard laboratory protocol. Demographic details and risk factors were noted from case records and correlated with Candida score.

Results

One hundred twenty-five non-duplicate samples (120 patients) positive on culture were included in the study. The most common co-morbid condition associated with fungemia was diabetes mellitus. The most common risk factor was total parenteral nutrition. Non-albicansCandida(*C. tropicalis*) was predominant. Candida species showed good sensitivity to voriconazole (80%) followed by fluconazole (67.78%) and amphotericin (62.22%). Twenty-nine patients had a Candida score of more than three.

Conclusion

Fluconazole available in both oral and parenteral formulations is an effective antifungal agent against the candida spp. Voriconazole should be reserved for non-responders. Rising resistance to common antifungals among *Candida albicans* is a matter of concern.

## Introduction

Fungi emerged as a major public health problem in early 1980. They are responsible for a significant increase in morbidity and mortality in intensive care units (ICU) and among immunocompromised patients [1]. Sepsis-induced mucosal or cutaneous barrier disruption, defects in production or impaired function of neutrophils, deranged cell-mediated immunity, metabolic dysfunction, and extremes of age circumvent the body’s defence mechanism, increasing the risk of fungal infection. Other factors that can jeopardize immunity are prolonged surgeries, protracted use of broad-spectrum antibiotics, cytotoxic chemotherapies, use of immunosuppressants in transplantation, intravenous nutrition, the use of multiple-lumen catheter, renal replacement therapy, and mechanical ventilation [2]. Only 5% of hospital beds are ICU beds, but ICU patients account for more than 20% of hospital-acquired infections [3].

Candida species are responsible for the majority of hospital-acquired fungal infection [4]. Centers for Disease Control (CDC) has listed *Candida albicans* as the seventh most common nosocomial pathogen [5]. According to the National Nosocomial Infection Surveillance (NNIS) system of the United States, it ranks fourth in causing nosocomial bloodstream infections (BSI) [6] and is associated with increased hospital stay and increased mortality.

A global rise in the incidence of invasive candidiasis (since the early 1990s) has been documented. National data suggests a relative increase in the proportion of non-albicans Candida (NAC) isolates like *Candida glabrata, Candida parapsilosis, Candida tropicalis, and Candida krusei* [7]. They are virulent and often associated with treatment failures.

India, being a tropical country, experiences a high prevalence of invasive candidiasis. Contributory factors include: favourable climatic conditions (warm and humid), large population of immuno-compromised hosts (high prevalence of diabetes, autoimmune disease, poor nutrition, retroviral infection) and over-the-counter access to antibiotics and steroids [8].

Diagnosing invasive candidiasis in the early stages entails a high index of suspicion. Clinical presentation is often non-specific and overlaps with those of bacterial infection [9]. Moreover, the major criteria for introducing empirical antifungal therapy in critically ill individuals are not well defined. Early use of antifungal therapy is not recommended [10]. It increases the cost of treatment, results in drug toxicities and emergence of drug resistance. However, antifungals are frequently used in ICU, in patients not responding to antibacterial therapy [11].

In 2009, a Spanish group, Leon et al., developed the “Candida Score” to predict invasive candidiasis. It was developed using the database of the Estudio de Prevalencia de Candidiasis project (Spanish acronym for Candidiasis Prevalence Study). Risk factors to predict invasive candidiasis included surgery, multifocal candida colonization, total parenteral nutrition (TPN) and severe sepsis [12].

Use of antifungal agents for prophylaxis in suspected systemic and invasive candidiasis (polyenes, allylamines, azoles, and the recently developed echinocandins) is reserved due to their adverse side effects, toxicity, and drug resistance. Fluconazole is most commonly used, as it is readily available and is economical [13].

Resistance to antifungal agents is on the rise [14]. The potential clinical importance of species-level identification has also been recognized. Candida species are known to differ in expression of various virulence factors and antifungal susceptibility [15]. The rising incidence of NAC isolates in the immunocompromised patients and the emergence of antifungal resistance, necessitates the judicious use of antifungal prophylaxis. Characterization and sensitivity profiles of locally prevalent Candida strains and the knowledge of risk factors for invasive candidiasis can help in deciding clinical strategies.

The current study was undertaken to characterize the Candida species in clinical specimens of patients admitted in the ICU, perform antifungal susceptibility tests, and correlate with Candida score to assess the need of antifungal treatment.

## Materials and methods

We conducted a prospective observational study over a period of 16 months, i.e., September 2018 to December 2019 in a tertiary care 500 bedded government institute. Approval of the Institutional Ethics Committee was obtained prior to the commencement of this study (459/IEC/2018/IGIMS). Relevant clinical samples depending on the possible source of infection (urine, sputum, blood, tracheal aspirate and urinary catheter) were collected following standard guidelines. Samples which grew yeast-like cells were considered positive. Following characteristics were recorded: age, gender, co-morbid illness (diabetes, tuberculosis, heart disease, renal failure, encephalopathy, anaemia), surgical intervention, features of sepsis and use of total parenteral nutrition.

Direct microscopic examination using the recommended KOH (potassium hydroxide) mount preparation was performed for specimens like sputum and urine sediment. Preparation was evaluated using a light microscope for the presence of yeast-like cells (YLC), with or without pseudo-hyphae. Smears (primary smear) were also examined after Gram staining, to look for the presence of Gram-positive budding YLCs.

Fungal culture was performed using blood agar and Sabouraud's Dextrose Agar (SDA) with antibiotics. The plates were incubated at 37°C and examined every other day for growth, up to 4-6 weeks. Colony characters, surface textures and pigmentation if any, were noted. Any creamy, smooth, pasty and convex colonies on SDA were subjected to Grams staining. Candida appears as a large (3-5 µm), Gram-positive budding yeast cell. From such colonies further speciation was done by germ tube formation, chlamydospore formation test using Cornmeal agar, CHROM agar test (M1297A HIMEDIA), carbohydrate utilization test, sugar assimilation test, urease test, nitrate assimilation test, growth at 450C and confirmed by automated identification system (VITEK 2 from Biomerieux using VITEK® 2 YST ID cards; Biomerieux, Marcy-l'Étoile, France).

Germ tube method

A 0.5 ml of pooled human serum was taken in a sterile test tube and inoculated with a colony of the yeast. The test tube was incubated at 370C for 2-4 hours after which, a drop of serum was transferred on a clear, sterile glass slide, covered with glass cover-slip and observed microscopically under low power objective. The presence of five or more germ tubes in the entire wet mount preparation was considered significant for *C. albicans *or* C. dubliniensis.*

Antifungal susceptibility testing (AFST) was done using the disc diffusion technique for seven antifungal agents (voriconazole, fluconazole, amphotericin B, itraconazole, ketoconazole, miconazole and nystatin).

Procedure of AFST

According to the Clinical and Laboratory Standards Institute (CLSI) document M44-A2, which is validated only for azoles and echinocandins against *Candida spp*., Mueller Hinton Agar (MHA) (HiMedia, Mumbai, India) was prepared as per the manufacturer’s instruction and supplemented with 2% glucose and 0.5 µg/ml Methylene blue dye. About five colonies were picked up and suspended in 5 ml of sterile saline. The suspension was vortexed and turbidity adjusted to 0.5 McFarland standard. After about 15-20 minutes of incubation, lawn culture was done using a sterile swab dipped in the suspension. Discs were dispensed and pressed onto the surface of the inoculated MHA plate after a waiting period of 15 minutes for the excess moisture to be absorbed. They were placed no less than 10 mm from the edge of the petri dish. Plates were inverted and placed in an incubator set at 35°C (± 2°C). Examination of the plates was done at 24 to 48 hours, depending on the growth rate of Candida species. The test organisms were categorized into different groups depending on the zone size against different antifungal agents. The zone diameter interpretation criteria classify the isolate into three categories: susceptible, susceptible dose-dependent, and non-susceptible/resistant [16].

The “Candida score” was calculated for all the patients as proposed by Leon et al. in 2009. Clinically severe sepsis was given a score of 2 and the rest risk factors (surgery, multifocal candida colonization, total parenteral nutrition) a score of 1. Cut-off value of 3 was considered significant [12].

## Results

During the study period (16 months), a total of 2129 non-duplicate samples were collected from ICUs, out of which 125 (120 patients) were found to be positive on microscopy and culture (both blood agar and SDA) for Candida species.

Of the 120 study cases, 64 (53.33%) were males. The most common co-morbidity associated with invasive candidiasis was diabetes and the most common risk factor according to the Candida score in the present study was the use of total parenteral nutrition (TPN) (n=29, 24.17%) (Table 1).

**Table 1:** Co-morbid conditions and risk factors associated with Candida infections

	Co-morbid conditions		Number of patients (%)
1	Respiratory distress		14 (11.66)
2	Acute renal failure (with/without sepsis)		15 (12.50)
3	Tuberculosis (pulmonary/extra pulmonary)		20 (16.67)
4	Diabetes mellitus (with complications)		35 (29.17)
5	Cerebral malaria		10 (8.33)
6	Alcoholic liver disease/hepatic encephalopathy		05 (4.17)
7	Postoperative state		09 (7.50)
8	Seizures		04 (3.33)
9	Multiple infarct/Heart disease		06 (5.00)
10	Anaemia		02 (1.67)
Risk factors		No. of patients	
Sepsis or SIRS		15 (12.50%)	
Surgery		9 (7.50%)	
Total parenteral nutrition		29 (24.17%)	
Multifocal candida colonization		5 (4.17%)	

**Figure 1:**
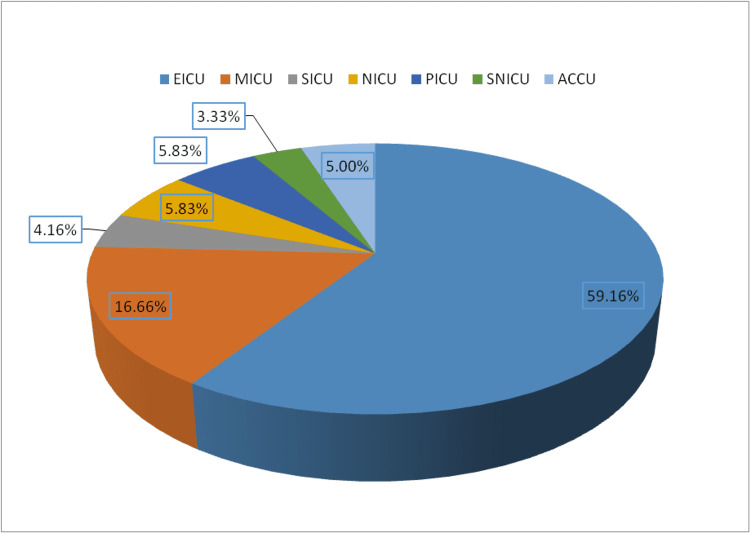
Intensive care unit wise distribution of patients with Candida isolates. ACCU: Advance cardiac care unit; SNICU: Surgical neonatal ICU; PICU: Pediatric ICU; NICU: Neonatal ICU; SICU: Surgical ICU; MICU: Medical ICU; EICU: Emergency ICU

The majority of samples (n=71, 59.16%) were from patients admitted in the emergency ICU (EICU) (Figure 1).

Among the 125 candida isolates from 120 patients, non-albicans Candida (NAC) species were predominant (n=90, 72.00%) and were isolated most commonly from urine (n=94, 75.20%), followed by blood (n=25, 20.00%) (Table 2).

**Table 2:** Sample-wise distribution of Candida species

Candida isolates	No. of isolates (%)	Urine (n=94)	Sputum (n=3)	Blood (n=25)	Urinary catheter (n=1)	ET tube aspirate (n=2)
Candida albicans	35 (28.00%)	30	02	02	0	01
Candida tropicalis	62 (49.60%)	47	01	14	0	0
Candida parapsilosis	13 (10.40%)	07	0	06	0	0
Candida glabrata	10 (8.00%)	06	0	02	01	01
Candida krusei	04 (3.20%)	03	0	01	0	0
Candida dubliniensis	01 (0.80%)	01	0	0	0	0

Bimodal distribution according to age was observed with majority either <10 years (n=19, 15.83%) or >50 years (n=51, 42.50%) of age (Table 3).

**Table 3:** Age-wise distribution of patients and Candida spp. isolates

Age group (Years)	*Candida albicans* (n=35)	*Candida tropicalis* (n=62)	*Candida parapsilosis* (n=13)	*Candida glabrata* (n=10)	*Candida krusei* (n=4)	*Candida dubliniensis *(n=1)
0-10 (n=19)	5	10	3	1	0	0
11-20 (n=12)	4	6	0	2	0	0
21-30 (n=11)	2	9	0	0	0	0
31-40 (n=16)	4	9	2	1	0	1
41-50 (n=15)	6	5	0	2	2	0
51-60 (n=18)	4	7	5	2	0	0
>60 (n=33)	10	16	3	2	2	0

Twenty-nine (24.16%) patients in our study cohort had a Candida score of ≥3 out of which severe sepsis was present in over half of the patients with a candida isolate. Total parenteral nutrition was responsible for one-third of patients (Table 4).

**Table 4:** Candida score variables in patients with score ≥ 3

Variables for Candida score (n=29)	No. of patients
Severe sepsis	15 (51.72%)
TPN (total parenteral nutrition)	9 (31.03%)
Surgery on ICU admission	6 (20.69%)
Multifocal Candida species colonization	5 (17.24%)

On in-vitro antifungal susceptibility testing, the majority of *Candida albicans *isolates were sensitive to voriconazole (71.42%, n=25) followed by amphotericin B (62.85%, n=22) and fluconazole (57.14%, n=20). Nearly three-fourths showed dose-dependent sensitivity to nystatin. A significant number of isolates were resistant to miconazole (51.42%, n=18) and ketoconazole (37.14%, n=13) (Table 5).

**Table 5:** Antifungal susceptibility pattern of Candida albicans (n=35 isolates)

Antifungal agents	Sensitive (%)	Resistant (%)	Dose Dependent (%)
Voriconazole	25 (71.42)	10 (28.57)	0 (0.00)
Fluconazole	20 (57.14)	12 (34.28)	3 (8.57)
Amphotericin B	22 (62.85)	8 (22.86)	5 (14.28)
Itraconazole	15 (42.86)	4 (11.42)	16 (45.71)
Ketoconazole	9 (25.71)	13 (37.14)	13 (37.14)
Miconazole	9 (25.71)	18 (51.42)	8 (22.86)
Nystatin	3 (8.57)	6 (17.14)	26 (74.28)

Non-albicans Candida (NAC) isolates showed a similar pattern. Eighty percent of the isolates were sensitive to voriconazole and nearly two-thirds to amphotericin B and fluconazole. Majority were resistant to miconazole (65.56%, n=59) and ketoconazole (46.66%, n=42). Dose-dependent sensitivity against nystatin was observed in 67.78% (61/90) (Table 6).

**Table 6:** Antifungal susceptibility pattern of non-albicans Candida (n=90 isolates)

Antifungal agents	Sensitive (%)	Resistant (%)	Dose Dependent (%)
Voriconazole	72 (80.00)	18 (20.00)	0 (0.00)
Fluconazole	61 (67.78)	7 (7.78)	22 (24.44)
Amphotericin B	56 (62.22)	22 (24.44)	12 (13.33)
Itraconazole	36 (40.00)	14 (15.56)	30 (33.33)
Ketoconazole	19 (21.11)	42 (46.66)	29 (32.22)
Miconazole	12 (13.33)	59 (65.56)	19 (21.11)
Nystatin	13 (14.44)	16 (17.78)	61 (67.78)

## Discussion

Intensive care units (ICUs) experience a high rate of nosocomial infections and associated morbidities. Incidence of ICU infections varies from 2-49% across various centres [17] which are frequently linked to rapidly emerging antimicrobial resistance affecting healthcare at the individual (patient) as well as community level. *Candida spp*. is capable of causing a wide range of superficial and deep-seated mycoses. Accurate identification of species and knowledge of sensitivity patterns dictate the timely initiation of appropriate antifungals and treatment outcomes. In the present study, fungal isolates were more commonly recovered among males (53.33%) consistent with studies by Meena et al., Kaur et al. and Rodriguez et al. [18-20]. It may be related to an increased incidence of catheter-related urinary infection among males.

The last two decades have witnessed an increase in fungal isolates from ICU subjects due to improvement in diagnostics, overuse of antibiotics and better organ support systems prolonging ICU stay. In a report from eastern India [21], Candida species were detected in nearly 15% of isolates.

More than one-fourth of patients detected with candidemia were more than 60 years of age, similar to a large European study by Tortorano et al. (>28% candidemia in subjects over 70 years of age) [22] and a Brazilian study by Resende et al. [23]. This possibly reflects the compromised immune system in this population or prolonged ICU stays [20]. Candida infection is not only related to the virulence of the pathogen, but also to the collapse in the defence mechanism of the host. Age-associated co-morbidities increase the chances of fungal invasion. In the present study, diabetes mellitus was present in nearly 30% of patients, followed by renal impairment (12.50%). Poor glycemic control and requirement of renal support system are associated with increased use of antibiotics, prolonged hospital stay, and thus, risk of acquiring candidemia [24].

The data herein demonstrates frequent infection with NAC (72.00%) (most commonly *Candida tropicalis*) as compared to *Candida albicans* (28%) (Table 2). This shift may be due to the increased use of antifungal drugs in hospital setups which resulted in the occurrence of resistant strains from NAC. *Candida parapsilosis* which has the tendency to cause device-related infections was isolated in nearly 10% [25].

Overall in-vitro susceptibility test findings were comparable to reports from other centres across the country (Tables 7, 8) [26-28]. Isolates from both the groups showed maximum sensitivity to Voriconazole (69-100%), fluconazole (36-100%) and amphotericin B (46-80%). A high incidence of drug-resistant Candida species was evident in our research, wherein isolates of both *Candida albicans* and NAC showed maximum resistance to miconazole (51.42%, 65.56%, respectively) and ketoconazole (37.14%, 46.66%, respectively) (Tables 5, 6). In-vitro resistance against fluconazole was alarmingly high among *Candida albicans* (34.28%) when compared to NAC isolates (7.78%) in the present study. However, a report from China [28] demonstrated higher resistance to fluconazole (72.19%) among NAC isolates. We observed high dose-dependent sensitivity for nystatin (74.28% for Candida species and 67.78% for NAC isolates) which implies that a low concentration of the drug may result in treatment failure in case of oral or gastrointestinal infections (Tables 5, 6). Nystatin can be effectively used against the dose-dependent multidrug-resistant species.

**Table 7:** Comparative data: Susceptibility pattern of Candida albicans to antifungal drugs

		Voriconazole	Fluconazole	Amphotericin B	Itraconazole	Nystatin
Present study (n=35)	Sensitive	25 (71.42%)	20 (57.14%)	22 (62.85%)	15 (42.85%)	3 (8.57%)
	Resistant	10 (28.57%)	12 (34.28%)	8 (22.85%)	4 (11.42%)	6 (17.14%)
Bhattacharjee [26] (2016) (n=28)	Sensitive	25 (89.28%)	28 (100.00%)	13 (46.42%)	22 (78.57%)	-
	Resistant	3 (10.71%)	0 (0.00%)	15 (53.57%)	6 (21.42%)	-
Pramodhini et al. [27] (2021) (n=10)	Sensitive	10 (100.00%)	10 (100.00%)	8 (80.00%)	-	-
	Resistant	0 (0.00%)	0 (0.00%)	2 (20.00%)	-	-
Wang et al. [28] (2016)	Sensitive	-	284/405 (70.12%)	-	151/387 (39.01%)	405/405 (100.00%)
	Resistant	-	36/405 (8.88%)	-	228/387 (58.91%)	0/405 (0.00%)

**Table 8:** Comparative data: Susceptibility pattern of non-Candida albicans to antifungal drugs

		Voriconazole	Fluconazole	Amphotericin B	Itraconazole	Nystatin
Present study (n=90)	Sensitive	72 (80.00%)	61 (67.77%)	56 (62.22%)	36 (40.00%)	13 (14.44%)
	Resistant	18 (20.00%)	7 (7.77%)	22 (24.44%)	14 (15.55%)	16 (17.77%)
Bhattacharjee [26] (2016) (n=36)	Sensitive	25 (69.44%)	13 (36.11%)	25 (69.44%)	18 (50.00%)	-
	Resistant	9 (25.00%)	22 (61.11%)	11 (30.55%)	14 (38.88%)	-
Pramodhini et al. [27] (2021) (n=60)	Sensitive	50 (83.33%)	41 (68.33%)	35 (58.33%)	-	-
	Resistant	10 (16.66%)	19 (31.66%)	25 (41.66%)	-	-
Wang et al. [28] (2016)	Sensitive	-	38/410 (9.26%)	-	136/401 (33.91%)	419/421 (99.52%)
	Resistant	-	296/410 (72.19%)	-	254/401 (63.34%)	2/421 (0.47%)

Resistance to azoles is of great concern as it is commonly used (apart from echinocandins) in the treatment of ICU patients. Fluconazole is available in both intravenous and oral formulations, has high bioavailability and the effect is dose dependent. It is cost-efficient in comparison to other antifungals and is well-tolerated. Though amphotericin B is effective against *Candida* strains, it is not the first choice treatment for candidemia due to significant nephrotoxicity [29].

Clinical manifestations in infection caused by *Candida albicans* are indistinguishable from those caused by NAC species but they differ in the expression of virulence factors, antifungal susceptibility and often demonstrate differential sensitivity to commonly used antifungals [27]. Therefore, it is important to identify candida to species level as well as perform assessing drug susceptibility tests. Data from one’s current institution will be helpful in managing fungal infections especially in critically ill patients [30]. Conventional methods used in routine laboratories for detecting fungal infections are based on morphology and physiology. However, identification of Candida up to species level necessitates a cassette of tests.

Candida score is an effective guide to the initiation of antifungals in non-neutropenic patients. Leon et al. in a multicentre study documented that invasive candidiasis was highly improbably if the Candida score in candida colonized critically ill patient is <3 [12]. In the present study, the score was ≥ 3 in only one-fourth of patients with a positive fungal culture. Severe sepsis (51.72%) and use of TPN (31.03%) were the most commonly associated risk factors. In the majority of patients with candidemia, the risk score was <3 and did not warrant the start of antifungals.

There are a few limitations of our study. It is limited to experience at a single institution. There is a scarcity of data in terms of prior antibiotic therapy, previous exposure to antifungal agents, and virulence associated with Candida species.

## Conclusions

The increasing population of immunocompromised patients, together with the rising incidence of NCA species and the emergence of acquired antifungal resistance, necessitates the judicious administration of antifungal prophylaxis in at-risk patients and empirical antifungal therapy in patients suffering from candidiasis. Characterization and sensitivity profiles of the locally prevalent Candida strains and knowledge regarding risk factors are essential for developing local guidelines based on disease epidemiology in the area. It will also help to bring down morbidity and mortality of patients as well as the overall treatment cost.
